# GABA_B_ Receptors Augment TRPC3-Mediated Slow Excitatory Postsynaptic Current to Regulate Cerebellar Purkinje Neuron Response to Type-1 Metabotropic Glutamate Receptor Activation

**DOI:** 10.3390/cells7080090

**Published:** 2018-07-29

**Authors:** Jinbin Tian, Michael X. Zhu

**Affiliations:** Department of Integrative Biology and Pharmacology, McGovern Medical School, The University of Texas Health Science Center at Houston, Houston, TX 77030, USA; jin.bin.tian@uth.tmc.edu

**Keywords:** transient receptor potential, TRPC3, mGluR1, GABA_B_, EPSC, Purkinje cell, cerebellum

## Abstract

During strong parallel fiber stimulation, glutamate released at parallel fiber-Purkinje cell synapses activates type-1 metabotropic glutamate receptor (mGluR1) to trigger a slow excitatory postsynaptic current (sEPSC) in cerebellar Purkinje neurons. The sEPSC is mediated by transient receptor potential canonical 3 (TRPC3) channels. Often co-localized with mGluR1 in Purkinje neuron dendrites are type B γ-aminobutyric acid receptors (GABA_B_Rs) that respond to inhibitory synaptic inputs from interneurons located in the molecular layer of cerebellar cortex. It has been shown that activation of postsynaptic GABA_B_Rs potentiates mGluR1 activation-evoked sEPSC in Purkinje cells, but the underlying molecular mechanism remains elusive. Here we report that the augmentation of mGluR1-sEPSC by GABA_B_R activation in Purkinje neurons is completely absent in TRPC3 knockout mice, but totally intact in TRPC1-, TRPC4-, and TRPC1,4,5,6-knockout mice, suggesting that TRPC3 is the only TRPC isoform that mediates the potentiation. Moreover, our results indicate that the potentiation reflects a postsynaptic mechanism that requires both GABA_B_Rs and mGluR1 because it is unaffected by blocking neurotransmission with tetrodotoxin but blocked by inhibiting either GABA_B_Rs or mGluR1. Furthermore, we show that the co-stimulation of GABA_B_Rs has an effect on shaping the response of Purkinje cell firing to mGluR1-sEPSC, revealing a new function of inhibitory input on excitatory neurotransmission. We conclude that postsynaptic GABA_B_Rs regulate Purkinje cell responses to strong glutamatergic stimulation through modulation of mGluR1-TRPC3 coupling. Since mGluR1-TRPC3 coupling is essential in cerebellar long-term depression, synapse elimination, and motor coordination, our findings may have implications in essential cerebellar functions, such as motor coordination and learning.

## 1. Introduction

Purkinje cells in the cerebellum serve vital functions in motor coordination and motor learning. These neurons receive glutamatergic inputs at their extremely elaborated dendrites from parallel fibers at the molecular layer of cerebellar cortex and send outputs to deep cerebellar nuclei in the form of γ-aminobutyric acid (GABA) [1,2]. The excitatory postsynaptic potentials or currents (EPSPs or EPSCs) elicited by the glutamatergic inputs in Purkinje neurons include both fast and slow components, which have been referred to as fast EPSC (fEPSC) and slow EPSC (sEPSC) in the case of voltage-clamp recordings [3]. While fEPSC is mainly mediated by the ionotropic glutamate receptors, sEPSC is triggered by the activation of metabotropic glutamate receptors (mGluRs), mainly the type 1 mGluR (mGluR1) [4]. Importantly, sEPSC only develops when the Purkinje cell receives a strong glutamate input, which in experimental settings requires multiple pulses of high intensity and high frequency electrical stimulations of the parallel fibers [3,5]. This reflects high levels of glutamate release at the parallel fiber-Purkinje cell synapses, causing a spillover of the neurotransmitter from the synaptic clefts to activate mGluR1 abundantly present at the extra-postsynaptic sites of these synapses [6]. mGluR1 is highly expressed in the brain, particularly Purkinje cells [7,8]. The mGluR1-mediated signaling is essential for synaptic plasticity of Purkinje cells, such as cerebellar long-term depression (LTD), the long-lasting reduction of transmission efficiency at the parallel fiber-Purkinje cell synapses [9]. The activation of mGluR1 triggers sEPSC, which is exclusively mediated by channels made of the transient receptor potential canonical 3 (TRPC3) protein [3]. 

TRPC3 is a member of the TRPC subgroup of transient receptor potential (TRP) superfamily of cation channels. The seven members of the TRPC subgroup (TRPC1-7) function as receptor-operated, non-selective cation channels activated downstream from the stimulation of phospholipase C (PLC) [10,11]. Since mGluR1 is coupled to G_q/11_ proteins which exert effects through PLCβ isoforms, it is natural for TRPC channels to be a part of signaling cascades associated with mGluR1 activation. Among all TRPCs, TRPC3 exhibits the highest expression in cerebellum, particularly Purkinje neurons [3,4], being most abundant in the somatodendritic compartment of these neurons [3]. Disruption of TRPC3 expression not only eliminated sEPSC in Purkinje neurons [3] but also impaired the normal cerebellar LTD induction [12]. Moreover, multiple motor deficits were observed in mice with an altered expression of TRPC3 or ablation of the *Trpc3* gene [4]. Therefore, by mediating sEPSC TRPC3 plays an important role in cerebellar function.

In addition to mGluR1, metabotropic GABA_B_ receptors (GABA_B_Rs) are also abundantly expressed at the extra-postsynaptic sites of the parallel fiber-Purkinje cell synapses [13,14,15]. It has been reported that in the continued activation of GABA_B_Rs, mGluR1 agonist-evoked inward current, representing sEPSC, in cerebellar Purkinje cells is augmented [5]. However, it is not known whether the enhanced response to mGluR1 stimulation resulted from potentiation of the mGluR1-TRPC3 coupling or recruitment of a new channel(s); especially given that unlike mGluR1, GABA_B_Rs are coupled to G_i/o_ proteins. In the present study, we used whole-cell patch clamp recordings to examine the receptors and ion channels that underlie the potentiation of mGluR1-sEPSC by GABA_B_Rs at the parallel fiber-Purkinje cell synapses. We found that GABA_B_R activation still converges on mGluR1-TRPC3 coupling to augment sEPSC and this modulation helps shape the Purkinje cell firing response to mGluR1 activation. 

## 2. Materials and Methods

### 2.1. Animals and Brain Slice Preparation

All animal procedures are approved by the Animal Welfare Committee of the University of Texas Health Science Center at Houston in accordance with NIH guidelines. TRPC3 knockout mice were generously provided by Dr. Oleh M. Pochynyuk at The University of Texas Health Science Center at Houston. TRPC1 knockout mice and TRPC1,4,5,6 quadruple knockout mice were generously offered by Dr. Lutz Birnbaumer at the National Institute of Environmental Health Sciences, USA. TRPC4 knockout mice were generously provide by Dr. Marc Freichel at Saarland University, Germany. The TRPC knockout mice are either in C57BL/6 background or mixed C57BL/6 × 129/Sv background. The wild-type (C57BL/6) and TRPC knockout mice used in this study were between postnatal days 17–22. Both male and female mice were used and no sex differences were found in the results. Mice were anesthetized by isoflurane before they were sacrificed. Whole cerebellum was excised quickly and immediately immersed into ice-cold, oxygenated artificial cerebrospinal fluid (aCSF, in mM): 125 NaCl, 26 NaHCO_3_, 2.5 KCl, 1.25 NaH_2_PO_4_, 1 MgCl_2_, 2 CaCl_2_, and 10 glucose, bubbled with 5% CO_2_ and 95% O_2_ (pH 7.4). Sagittal cerebellar slices of 300 μm thickness were prepared from vermis of the cerebellum with a vibratome (VT1200S, Leica Biosystems, Wetzlar, Germany) in ice-cold, oxygenated aCSF. Slices were recovered at 35 °C for 1 h and then maintained at room temperature (22–24 °C) in aCSF until use. 

### 2.2. Drug Delivery

**Figure 1:**
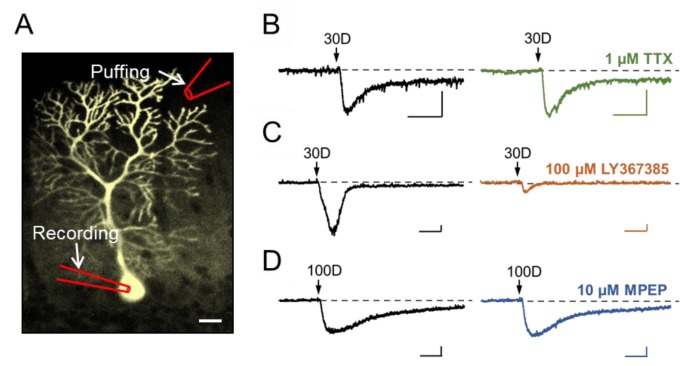
Activation of postsynaptic mGluR1 by a brief ejection of DHPG to dendrites triggers sEPSC in cerebellar Purkinje neurons. (**A**) Fluorescent image of a Purkinje cell loaded with Lucifer Yellow through the recording pipette placed at soma. The location of the puffing pipette for drug ejection is shown on the top. Scale bar: 50 μm. (**B**–**D**) Consecutive recordings of sEPSCs evoked by DHPG (30 or 100 μM, shown as 30D or 100D, respectively) from individual Purkinje cells before (left) and after (right) application of 1 μM TTX (B), 100 μM LY367385 (C), or 10 μM MPEP (D) through whole-chamber perfusion. Representative of 2–4 cells with similar results. Arrows point to the time when DHPG was ejected. Scale bars: 1 s, 100 pA.

Each cerebellar slice placed in the recording chamber was continuously perfused with the normal aCSF unless indicated otherwise. Drugs were diluted in aCSF and applied either through whole-chamber perfusion or by pressure ejection via a drug-delivery glass pipette positioned about 300 μm away from the targeted cell in the molecular layer of the cerebellar cortex (Figure 1A). The pressure ejections (puffs) were delivered by TooheySpritzer pressure system IIe (Toohey Company, Fairfield, NJ, USA) with the trigger controlled via stimulation protocols programmed using PatchMaster software (HEKA Instruments, Holliston, MA, USA). The puff duration was 100–200 ms and the air pressure ranged 5–20 psi. The recording chamber was continuously perfused with aCSF during the drug ejection.

### 2.3. Drugs

(*S*)-3,5-Dihydroxyphenylglycine (DHPG), LY367385, 2-methyl-6-(phenylethynyl)pyridine hydrochloride (MPEP), (R)-baclofen and CGP 55845 were purchased from Abcam Biochemicals (Cambridge, MA, USA). Tetrodotoxin (TTX) was purchased from Tocris Bioscience (Minneapolis, MN, USA). GSK2293017A was a kind gift from Prof. Xuechuan Hong (Wuhan University, China).

### 2.4. Electrophysiology

Glass pipettes (Sutter Instrument, Novato, CA, USA) were pulled using a Narishige PC-10 puller (Narishige International USA, Amityville, NY, USA). Whole-cell patch clamp recordings were made using pipettes with tip resistance of 3–6 MΩ when filled with internal solution containing (in mM): 130 K-methanesulfonate, 7 KCl, 0.05 EGTA, 1 Na_2_-ATP, 3 Mg-ATP, 0.05 Na_2_-GTP and 10 HEPES, with pH adjusted to 7.3 by KOH and osmolarity of 300 mOsm. Purkinje cells (lobules V-VII) were visualized using a 60x water objective lens and infrared-differential interference contrast videomicroscopy (Olympus BX51WI with OLY-150IR video camera, Olympus America, Center Valley, PA, USA). sEPSC was recorded in voltage-clamp mode when the cell was held at −70 mV. Membrane potential and action potential firing were recorded in current-clamp mode. Immediately before switching to current clamp, cells were held at −45 or −50 mV. After switching to current-clamp, a constant current based on the value in voltage-clamp mode was injected to maintain the membrane potential at the set level. Only Purkinje cells which showed tonic firing around −50 mV were included in the firing frequency analysis. The temperature of the recording chamber was maintained at approximately 32 °C by passing the perfusion solution (aCSF) through an in-line heater (Warner Instruments, Hamden, CT, USA) at 3 mL/min driven by a Rabbit^TM^ peristaltic pump (Mettler-Toledo Rainin, Oakland, CA, USA). 

### 2.5. Data Acquisition and Analysis

Data were acquired using an EPC10 amplifier operated by PatchMaster software (both from HEKA Instruments). Recordings were filtered at 2.9 kHz and digitized at 10 kHz. The amplitude of the sEPSC was measured at the time point when the inward current reached the maximum. All measurements were made off-line using the analysis function of the PatchMaster software. Data are presented as means ± SEM. Bar graphs were produced in GraphPad Prism 6 software (GraphPad software, Inc., San Diego, CA, USA). Differences are considered statistically significant when *P* < 0.05. 

## 3. Results

### 3.1. Stimulation of Dendritic mGluR1 Evokes an sEPSC-Like Inward Current in Cerebellar Purkinje Neurons

Previous studies have shown the critical involvement of TRPC3 in mediating sEPSC of cerebellar Purkinje cells in response to stimulation of mGluR1 [3,4]. To confirm this in mouse brain slices, we used a pressure-driven drug ejection (puffing) system to eject varying concentrations of the mGluR1 agonist, (*S*)3,5-dihydroxyphenylglycine (DHPG), towards the molecular layer of cerebellum, which consists of dendritic trees of Purkinje cells. The response of individual Purkinje cells to DHPG stimulation was recorded by whole-cell patch clamp technique from the soma of each neuron (Figure 1A). As shown in Figure 1B–D (left panel), with the cells held at −70 mV under voltage clamp, the ejection of DHPG (30–100 μM, 100–200 ms) evoked inward currents that rose slowly and lasted for >1 s. These represent sEPSC because of the much slower kinetics of onset and longer lasting duration than fEPSCs typically mediated by ionotropic glutamate receptors [3]. The DHPG-elicited inward current (herein referred to as sEPSC) is independent of neurotransmission, as it was not affected by blocking neurotransmitter release with whole-chamber perfusion of 1 μM tetrodotoxin (TTX) (Figure 1B). Supporting the involvement of mGluR1, but not mGluR5, the DHPG-elicited sEPSC was abolished almost completely by LY367385 (100 µM), a specific mGluR1 antagonist (Figure 1C), but not MPEP (10 µM), a specific mGluR5 antagonist (Figure 1D), both applied through whole-chamber perfusion. These results suggest that postsynaptic mGluR1 expressed in the dendrites of cerebellar Purkinje neurons mediated DHPG-evoked sEPSCs.

### 3.2. TRPC3 Mediates the mGluR1 Activation-Evoked sEPSC in Cerebellar Purkinje Neurons

**Figure 2:**
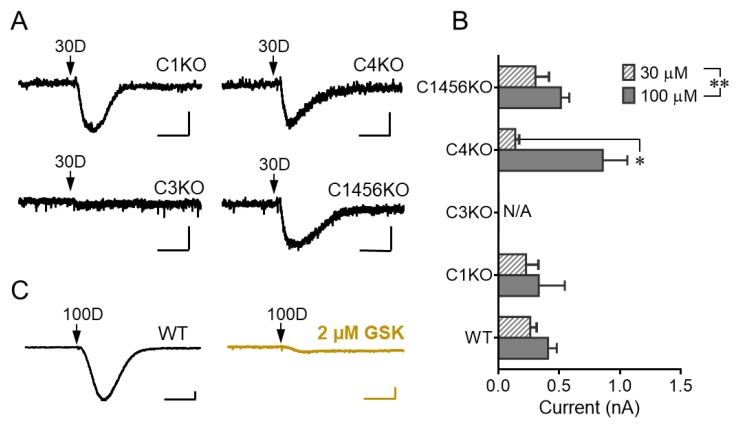
TRPC3 mediates DHPG-evoked sEPSC in mouse cerebellar Purkinje cells. (**A**) Representative current traces showing responses of cerebellar Purkinje cells in brain slices from different *Trpc* gene knockout mice to 30 μM DHPG (30D). C1KO, *Trpc1* knockout; C3KO, *Trpc3* knockout; C4KO, *Trpc4* knockout; C1456KO, *Trpc1,4,5,6* quadruple knockout. Note, only C3KO exhibited no response to DHPG. (**B**) Bar graph showing mean ± SEM of peak amplitudes of inward currents at −70 mV elicited by DHPG (30 and 100 µM) in Purkinje cells from wild type (WT) and *Trpc* knockout mice. *n* = 3–14 for each group. Two-way ANOVA analysis followed by Sidak post hoc test, * *P* < 0.05 between 30 and 100 μM in C4KO group, ** *P* < 0.01 for the factor of drug concentration, no significant difference for the factor of genotype. (**C**) Consecutive recordings of sEPSCs evoked by 100 µM DHPG before (left) and after (right) whole-chamber perfusion of 2 μM GSK2293017A in a Purkinje cell from wild type mouse. Representative of two cells with similar results. Arrows point to the time when DHPG was ejected. Scale bars: 1 s, 100 pA.

While the DHPG-evoked sEPSC in cerebellar Purkinje cells of adult mice has been shown to be mediated by TRPC3 [3], that in developing Purkinje neurons of young animals had been suggested to also depend on TRPC1 [16]. Given that the Purkinje cells also express other TRPC isoforms, albeit at lower levels than that of TRPC3 [4], we also evaluated the possible involvement of several TRPC isoforms in DHPG-evoked sEPSC under our experimental setting using transgenic mice with selective ablation of *Trpc* genes. With pressure ejection of 30 and 100 μM DHPG, we detected sEPSCs in cerebellar Purkinje neurons from brain slices prepared from *Trpc1* knockout (KO) mice, *Trpc4* KO mice, and *Trpc1,4,5,6* quadruple KO mice, but not that from *Trpc3* KO mice (Figure 2A,B), suggesting that among the 5 TRPC isoforms tested, only TRPC3 is involved in DHPG-elicited sEPSC response. For both DHPG concentrations, the mean amplitudes of the sEPSCs in all transgenic lines, except for the *Trpc3* KO mice, were not different from that of wild type mice (Figure 2B), confirming again the lack of contribution of TRPC1, C4, C5, and C6 to this response. Consistent with the absolute dependence on TRPC3, the strong sEPSC elicited by 100 µM DHPG in wild type Purkinje cells was abolished by whole-chamber perfusion of GSK2293017A (2 μM), a specific TRPC3/C6 antagonist [17] (Figure 2C). Taken together, these results confirm that TRPC3 is the sole TRPC isoform involved in mediating sEPSC in mouse cerebellar Purkinje neurons in response to DHPG stimulation of mGluR1 under our experimental conditions. 

### 3.3. Stimulation of GABA_B_R Potentiates mGluR1 Agonist-Evoked sEPSC in Cerebellar Purkinje Neurons via a Postsynaptic Mechanism

**Figure 3:**
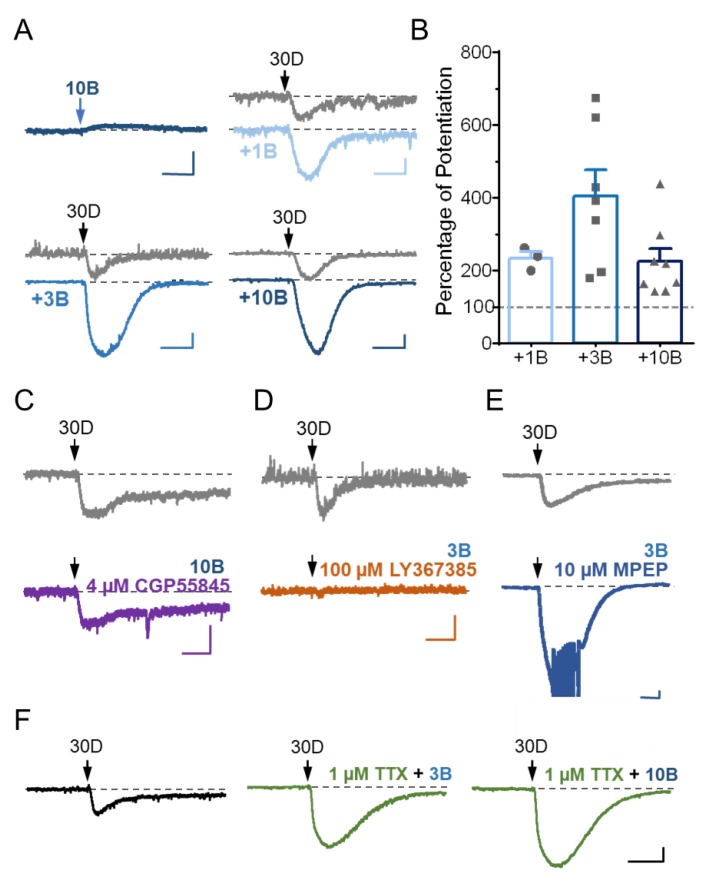
Stimulation of GABA_B_Rs potentiates DHPG-evoked sEPSC in Purkinje neurons by a postsynaptic mechanism. (**A**) Representative current traces showing responses of Purkinje cells to pressure ejection of 10 μM baclofen (10B) or the ejection of 30 μM DHPG (30D) before (upper) and after (lower) the application of 1, 3, and 10 μM baclofen (+1B, +3B, and +10B, respectively) through whole-chamber perfusion. (**B**) Bar graph showing the potentiation of 30 µM DHPG-evoked sEPSC by 1, 3 and 10 μM baclofen. Peak sEPSC amplitudes were normalized to that before baclofen application (set as 100% and shown by the dotted line). Shown are individual data points and summary (mean ± SEM). One-way ANOVA analysis followed by Sidak post hoc test, no significant differences among three baclofen concentrations. (**C**–**F**) Receptor types and postsynaptic mechanism of GABA_B_R action. Representative current traces showing responses of individual Purkinje cells to pressure ejection of 30 μM DHPG before and after application of 4 µM CPG55845 (C), 100 µM LY367385 (D), 10 µM MPEP (E), and 1 µM TTX (F) in combination with either 3 or 10 μM baclofen as indicated through whole-chamber perfusion. Representatives of 2–4 cells with similar results. For (**A**,**C**–**F**), arrows point to the time when baclofen or DHPG was ejected. Scale bars: 1 s, 100 pA.

Previously, the mGluR1 agonist-activated currents in Purkinje neurons were shown to be enhanced following the activation of GABA_B_Rs [5]. Different from mGluR1, which is coupled to G_q/11_-PLCβ signaling, the GABA_B_Rs are known to be preferentially coupled to pertussis-toxin sensitive G_i_ and G_o_ proteins [18]. Therefore, the co-stimulation of mGluR1 and GABA_B_Rs represents co-incident G_q/11_ and G_i/o_ signaling. To test if GABA_B_R signaling also affects the DHPG-evoked sEPSCs under our experimental setting, we used the GABA_B_R agonist, baclofen. Pressure ejection of baclofen (10 µM) alone onto the molecular layer of cerebellum in brain slices from wild type mice evoked a small and slowly developing outward current (Figure 3A), consistent with the idea that GABA_B_Rs are inhibitory because of their coupling to G_i/o_ proteins [18]. The baclofen-evoked outward current lasted as long as the drug was present and persisted for seconds even after the drug had been washed out (data not shown, but see [13]). In the continued presence of baclofen (1–10 µM, applied through whole-chamber perfusion), the ejection of 30 µM DHPG evoked larger sEPSC than in the absence of baclofen (Figure 3A,B), indicating that instead of being inhibitory, the activation of GABA_B_R actually enhances the excitatory response of mGluR1 activation by DHPG. On average, the whole-chamber perfusion of 1, 3, and 10 μM baclofen all potentiated sEPSC peak amplitude elicited by 30 μM DHPG by about double (Figure 3B), suggesting that either the potentiation only requires very weak GABA_B_R activation or it is easily saturable. Thus, consistent with the previous study [5], the mGluR1 agonist-induced sEPSC in cerebellar Purkinje cells is potentiated by the co-activation of GABA_B_Rs.

To confirm that baclofen indeed acted at the GABA_B_Rs to cause sEPSC potentiation in Purkinje neurons, we applied a specific GABA_B_R antagonist, CGP55845, together with baclofen. In the presence of 4 µM CPG55845, baclofen (10 µM) failed to enhance the DHPG-evoked sEPSC (Figure 3C). On the other hand, the co-application of the mGluR1 antagonist LY367385 (100 µM), but not that of the mGluR5 antagonist MPEP (10 µM), completely abolished the generation of sEPSC in response to 30 µM DHPG even in the presence of 3 µM baclofen (Figure 3D–E), suggesting that baclofen did not recruit a separate mGluR subtype(s) or another DHPG-sensitive receptor type(s) to trigger a new form of sEPSC. Therefore, the co-activation of GABA_B_Rs enhanced sEPSC triggered by the stimulation of mGluR1 in cerebellar Purkinje neurons.

Given that GABA_B_Rs are present at both the presynaptic and postsynaptic sides of parallel fiber-Purkinje cell synapses [5,13,14,15,19,20], the potentiation action of GABA_B_Rs on the DHPG-evoked sEPSC could arise either presynaptically or postsynaptically. To clarify these possibilities, we applied TTX (1 µM) to the brain slices through whole chamber perfusion. By blocking synaptic transmission, TTX should inhibit the presynaptic action of GABA_B_Rs on postsynaptic currents. However, in the presence of TTX, baclofen (3 and 10 μM) still markedly enhanced the current evoked by pressure ejection of 30 µM DHPG (Figure 3F), suggesting a postsynaptic mechanism for the potentiation of DHPG-evoked sEPSC by GABA_B_R activation in cerebellar Purkinje neurons. 

### 3.4. TRPC3 Underlies the Potentiation of sEPSC by GABA_B_R Stimulation

**Figure 4:**
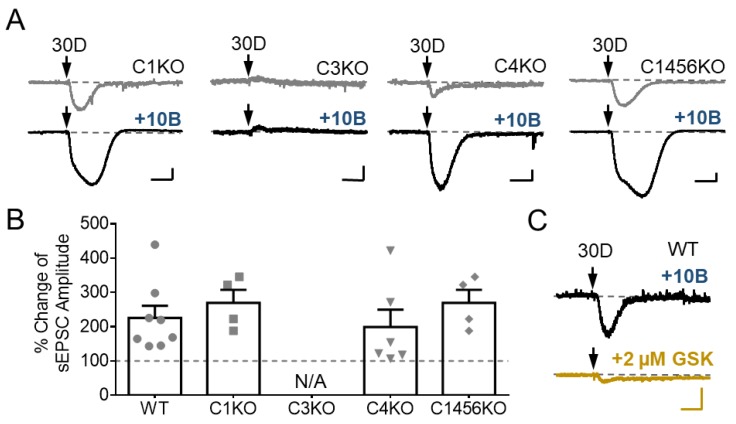
Baclofen potentiates TRPC3-mediated currents evoked by DHPG in cerebellar Purkinje neurons. (**A**) Representative current traces showing responses of cerebellar Purkinje cells in brain slices prepared from *Trpc*1 KO (C1KO), *Trpc3* KO (C3KO), *Trpc4* KO (C4KO), and *Trpc1,4,5,6* quadruple KO (C1456KO), to pressure ejection of 30 μM DHPG (30D) in the absence (upper) and presence of 10 μM baclofen (10B) applied through whole-chamber perfusion. Only C3KO neurons failed to develop the inward current in response to DHPG no matter baclofen was applied or not. (**B**) Bar graph showing the effect of 10 µM baclofen on sEPSCs elicited by ejection of 30 µM DHPG in wild type (WT) and *Trpc* KO Purkinje neurons. Peak sEPSC amplitudes were normalized to that before baclofen application (set as 100% and shown by the dotted line). The values for C3KO (*n* = 5) could not be calculated because the sEPSC amplitudes are all 0. Shown are individual data points and summary (mean ± SEM). One-way ANOVA analysis followed by Sidak post hoc test did not show significant differences among different genotypes. (**C**) GSK2293017A strongly inhibited sEPSC evoked by ejection of DHPG (30 µM) in the presence of baclofen (10 µM) in wild type Purkinje neurons. Representative current trace for three neurons. For (A,C), arrows point to the time when DHPG was ejected. Scale bars: 1 s, 100 pA.

The potentiation of DHPG-evoked sEPSC by the postsynaptic GABA_B_Rs could arise either from enhancing the activity of TRPC3, which underlies the DHPG-induced inward current in cerebellar Purkinje neurons, as shown earlier in Figure 2, or by recruiting other channels that respond specifically to mGluR1 and GABA_B_R co-activation. To test these possibilities, we examined the effect of baclofen on DHPG-evoked currents in Purkinje cells of brain slices prepared from *Trpc* gene KO mice. We found that while neurons from *Trpc1* KO, *Trpc4* KO, and *Trpc1,4,5,6* quadruple KO mice showed similar responses as the wild type neurons to DHPG and the potentiation by baclofen, those from *Trpc3* KO animals failed to develop inward currents in response to DHPG (30 µM) either in the absence or presence of baclofen applied through whole-chamber perfusion (Figure 4A,B). Furthermore, in wild type Purkinje neurons, the TRPC3 blocker, GSK2293017A, not only inhibited the DHPG-elicited sEPSC in the absence of baclofen (Figure 2C) but also abolished it in the presence of the GABA_B_R agonist (Figure 4C). Collectively, these results suggest an exclusive dependence on TRPC3 channels on not only the generation of sEPSC by mGluR1 agonist, but also its potentiation by postsynaptic GABA_B_R activation in cerebellar Purkinje neurons.

### 3.5. GABA_B_R Co-Stimulation Reshapes mGluR1-mediated Increase of Purkinje Cell Firing

Like in most brain areas, GABA is commonly considered an inhibitory neurotransmitter in the cerebellar cortex. Upon parallel fiber stimulation, GABA is released from interneurons that make synaptic connections with Purkinje cells. This would suppress the excitatory action of glutamate released by the parallel fiber at the parallel fiber-Purkinje cell synapse through activation of both ionotropic GABA_A_ receptors and metabotropic GABA_B_Rs. The GABA_B_R action can be both pre- and postsynaptic, as shown by the previous study [5]. Particularly, the presynaptic action of GABA_B_Rs is purely inhibitory, resulting in reduced glutamate release and thereby inhibition of both fEPSC and sEPSC. By contrast, at the postsynaptic level, GABA_B_R activation can assume both inhibitory or excitatory roles, with the latter being dependent on mGluR1 activation and the presence of TRPC3 channels. However, exactly how GABA_B_R potentiation of the mGluR1 activation-induced TRPC3 inward cation currents might modulate Purkinje cell function remained mysterious, as the previous study did not attempt to separate the pre- and post-synaptic functions of GABA_B_Rs by maintaining the constant stimulation levels of mGluR1 [5]. 

**Figure 5:**
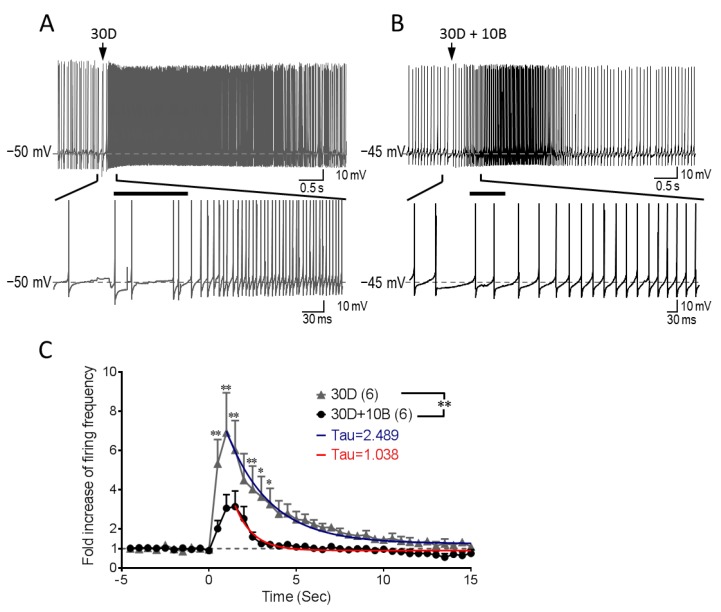
GABA_B_Rs attenuate mGluR1-TRPC3-mediated firing increase and facilitate firing recovery in Purkinje cell. Current-clamp recordings of membrane depolarization and action potential generation (firing) in response to pressure ejection of 30 µM DHPG (30D) alone (**A**) or co-ejection of 30 μM DHPG and 10 µM baclofen (30D + 10B) (**B**). Lower traces are expanded regions indicated between the two short vertical lines from the top traces. The initial holding potentials were −50 mV (A) or −45 mV (B). Arrows in the top traces point to the time when drugs were ejected. Horizontal bars in the lower traces mark the time period when drugs were ejected. (**C**) Purkinje cell instant firing frequency changes before and after drug treatments. Co-ejection of 10 µM baclofen significantly attenuated firing increase evoked by 30 µM DHPG in Purkinje cells. It also significantly accelerated the recovery of the firing to the original frequency. *n* = 6 for each group. Two-way ANOVA analysis followed by Holm-Sidak post hoc test, ** *P* < 0.01 between the two drug treatments; ** *P* < 0.01, * *P* < 0.05 at individual time points between two drug groups.

TRPC3 exerts a prominent effect on tonic firing frequency of Purkinje neurons [21]. Therefore, to address the functional significance of GABA_B_R potentiation of TRPC3 activity, we used current clamp recording to examine how Purkinje neuron firing is altered by activating TRPC3 through stimulation of mGluR1 in the absence and presence of GABA_B_R activation by baclofen. Purkinje cells in brain slices were initially held close to −50 mV, which produced spontaneous firing. When DHPG alone was applied by pressure ejection (100 ms), a significant increase of Purkinje cell firing was instantly initiated along with a small baseline membrane depolarization. It took about 1 s for the instant firing frequency to reach to a peak before the frequency winded down slowly with a time constant (Tau) of 2.5 s (Figure 5A,C). When DHPG was applied together with baclofen, increases in firing frequency were also observed. However, it took about 1.5 s for the instant firing frequency to achieve the maximum, which was less robust than that evoked by DHPG alone (Figure 5B,C). On the other hand, the decay of the firing frequency after reaching the peak was faster with the DHPG and baclofen co-stimulation, showing a time constant of 1.04 s. In appearance, such a negative impact of baclofen on mGluR1-TRPC3 mediated firing increase would be inconsistent with the strong potentiation of TRPC3 sEPSC by GABA_B_R stimulation observed in voltage-clamp recordings. However, given that the action potentials were recorded under the current-clamp configuration, in which voltage-gated channels are allowed to be activated, it is reasonable to assume that the stronger activation of TRPC3 was also accompanied with greater activations of Ca^2+^-activation and/or voltage-sensitive K^+^ channels that could more quickly dampen the depolarizing effect of TRPC3. Thus, these results suggest that the activation of GABA_B_Rs exerts a pronounced effect on shaping the TRPC3-mediated response of cerebellar Purkinje cells to mGluR1 activation. 

## 4. Discussion

Increasing evidence has implicated the importance of TRPC3 in cerebellar Purkinje cell function by mediating sEPSCs in response to stimulation of mGluR1 [3,4,12]. The TRPC3 activity accelerates the firing rate of Purkinje neurons [21]. Given that TRPC channels are generally activated downstream from receptors that signal through PLC [4], the functional coupling between mGluR1 and the TRPC3 channel in Purkinje neurons is not surprising. However, it is not known that TRPC3 is also regulated by G_i/o_ proteins although the G_i/o_-dependence has been demonstrated for TRPC4/C5 [22,23,24]. The current study suggests that endogenous TRPC3 channels in cerebellar Purkinje cells are regulated by G_i/o_ protein signaling, stimulated by postsynaptic GABA_B_Rs in these neurons. Our data demonstrate that GABA_B_R activation alone is insufficient to stimulate TRPC3, but when combined with mGluR1 activation, the GABA_B_R-evoked signaling not only markedly enhances TRPC3 currents, but also alters the response of Purkinje cell firing to mGluR1 stimulation. 

**Figure 6:**
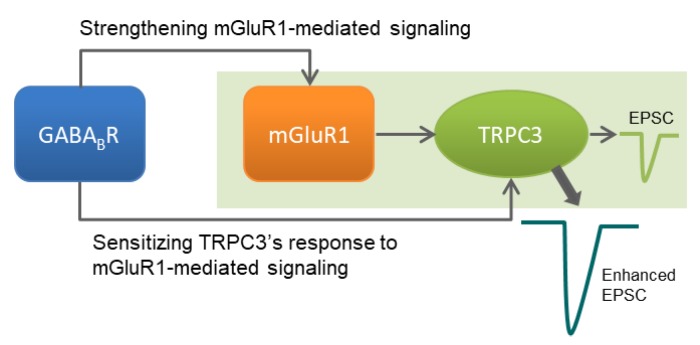
Diagram of GABA_B_R-mediated potentiation of mGluR1-TRPC3 signaling pathway in cerebellar Purkinje cells. Stimulation of mGluR1 at the dendrites of Purkinje cells activates TRPC3 channels to evoke an excitatory postsynaptic current (EPSC). The EPSC is enhanced if GABA_B_Rs expressed on the dendrites of the same Purkinje cell are activated at the same time. The enhanced EPSC is still mediated by mGluR1-TRPC3 coupling, presumably through enhancing mGluR1 function and/or increasing the sensitivity of TRPC3 to mGluR1 mediated signaling in Purkinje cells.

Previously, GABA_B_R activation has been shown to enhance mGluR1 agonist-evoked inward currents in cerebellar Purkinje neurons [5]. The mGluR1 agonist-evoked inward currents were later determined to be exclusively dependent on TRPC3, but not other TRPC isoforms [3], a conclusion supported by our current study (Figure 2). We further demonstrate that the GABA_B_R potentiation is also absolutely dependent on TRPC3 (Figure 4), ruling out the possibility that GABA_B_Rs recruit additional channel types that only respond to the co-stimulation of both mGluR1 and GABA_B_Rs. Thus, GABA_B_Rs exert their potentiation effect on sEPSC through TRPC3, the same channel that responds to mGluR1 activation. The potentiation effect of GABA_B_Rs was shown to be dependent on G_i/o_ signaling with the use of *N*-ethylmaleimide (NEM) in the previous study [5]. Surprisingly, the same study also suggested that the G_i/o_-dependent potentiation of the inward currents was specific for GABA_B_Rs, as stimulation of other G_i/o_-coupled receptors—such as acetylcholine, serotonin, and adenosine receptors—failed to mimic the effect of the GABA_B_R agonist [5]. Therefore, G_i/o_ protein signaling appears to be necessary but not sufficient for the potentiation of TRPC3-mediated sEPSC. It remains to be determined whether such specificity is a result of close colocalization between mGluR1 and GABA_B_Rs at the peripheral of parallel fiber-Purkinje cell synapse [5] or unique signaling pathway(s) activated by GABA_B_Rs but not other G_i/o_-coupled receptors. In fact, it has been reported that in cerebellar Purkinje cells, GABA_B_Rs constitutively enhance mGluR1 signaling in a manner that is dependent on the extracellular Ca^2+^ concentration, but independent of GABA_B_R-mediated cell signaling. This presumably occurs through a direct binding of extracellular Ca^2+^ to GABA_B_Rs, which alters the conformation of GABA_B_Rs and further modifies the direct interaction between GABA_B_Rs and mGluR1, leading to an enhanced function of mGluR1 [25]. Although co-immunoprecipitation experiments suggest that GABA_B_Rs and mGluR1 form physical association at the dendritic synapses of Purkinje cells and both mGluRs and GABA_B_Rs are atypical (Class C) G protein-coupled receptors that only work as dimers [26], to date, no evidence suggests that GABA_B_Rs oligomerize with mGluR1. Even if they do oligomerize, the oligomerized receptor complex would unlikely be responsive to the GABA_B_R agonist, as baclofen alone did not induce any inward current, meaning that without an mGluR1 agonist, the receptor complex could not trigger TRPC3 activation. It is possible that signaling pathway(s) downstream from GABA_B_Rs is involved, which acts either at the level of mGluR1 or that of TRPC3 (Figure 6). Indeed, mGluR1 may be sensitized by shifting to a high affinity binding state [27] and TRPC3 can be activated via binding of β-arrestin, one of the signaling pathways associated with the activation of G_i/o_-coupled receptors [28]. 

Our results indicate that TRPC3 expressed on Purkinje cell dendrites not only responds to postsynaptic mGluR1 activation with an inward cation current that induces membrane depolarization but also integrates the signals from G_i/o_-coupled GABA_B_Rs to shape the overall response of the Purkinje neuron to the co-transmission of the excitatory and inhibitory neurotransmitters, glutamate, and GABA, respectively. For the TRP channel, this appears to strengthen the channel activity, giving rise to potentiation of sEPSC; however, the net output of this integrated co-transmission is a better controlled time window of excitatory effect of mGluR1 activation on Purkinje neuron firing (Figure 5). The co-stimulation of GABA_B_Rs helps shape the mGluR1-mediated firing changes in tonic firing Purkinje neurons, by slowing down and dampening the mGluR1-mediated firing increase and accelerating the recovery of the augmented firing (Figure 5). These results appear to be inconsistent with the voltage-clamp recording data showing that GABA_B_Rs potentiate TRPC3 current (enhanced sEPSC). However, considering that membrane potentials are allowed to change freely under current-clamp but not voltage-clamp, the opposite net outcomes under the two recording modes are not unexpected. The voltage-gated K^+^ channels and Ca^2+^-activated and voltage-sensitive K^+^ channels, such as the large conductance Ca^2+^-activated K^+^ channels, are likely activated subsequent to TRPC3 and voltage-gated Ca^2+^ channels during action potentials. The activation of K^+^ channels counters the depolarization action of TRPC3. Depending on the types of K^+^ channels involved and their voltage and Ca^2+^ sensitivities, the net effect of TRPC3 activation can be depolarizing or hyperpolarizing, but it is predictable that when TRPC3 is strongly activated, more K^+^ channels will be recruited due to the very strong depolarization and Ca^2+^ influx, which will bring down the membrane potential. Therefore, TRPC3 plays a central role in mGluR1-mediated synaptic responses, which are further modulated by GABA input through GABA_B_Rs to shape Purkinje cell firing in the cerebellum. 

It is particularly worth noting that GABA_B_R co-stimulation with mGluR1 accelerated the recovery of Purkinje cell firing to the original frequency (Figure 5). Such a modification may be functionally relevant. Because of the slow development of sEPSC, as opposed to fEPSC mediated by ionotropic glutamate receptors, the termination of sEPSC is also slow, leading to slow recovery of the tonic firing to the original rate. This may be undesirable for the neuron as the sluggish recovery interferes with subsequent neurotransmission. Thus, the co-activation of GABA_B_Rs helps shut off the mGluR1 effect properly in Purkinje neurons. It remains to be clarified if this effect on termination is due to the hyperpolarization action of GABA_B_Rs through stimulating K^+^ channels or it is a result of TRPC3 channel potentiation. The outward current elicited by baclofen seen in the absence of mGluR1 activation represents K^+^ conductance [5], but the identity of the K^+^ channel(s) activated in response to GABA_B_R agonist in Purkinje neurons remains undefined. Although Purkinje cells express G protein-activated inwardly rectifying K^+^ (GIRK) channels, the GABA_B_R agonist-evoked outward current was unlikely mediated by GIRK, but instead might be attributable to Ca^2+^-activated K^+^ channels [5,18]. In such a case, the potentiated TRPC3 activity could have multiple ways to augment the activity of Ca^2+^-activated K^+^ channels. First, the enhanced Ca^2+^ influx through TRPC3 could increase the K^+^ channel activity. Second, the enhanced Na^+^ influx could induce stronger membrane depolarization, which could open more voltage-gated Ca^2+^ channels for more Ca^2+^ influx. Third, if the large conductance Ca^2+^-activated K^+^ channel, which is expressed in Purkinje cells, were involved, the membrane depolarization would also intensify its activity directly because of its voltage dependence. Finally, the rise of cytosolic Ca^2+^ also inhibits TRPC3 channel activity via a negative feedback mechanism [29,30]. Therefore, multiple mechanisms may act in concert to terminate the increase in Purkinje cell firing induced by mGluR1 activation in a timely manner. 

Purkinje neurons represent the sole output of the cerebellar cortex. Their function is important for various motor functions, including movement control, learning, and coordination. Both mGluR1 and TRPC3 have been clearly shown to be critical for motor function. In mice, genetic deletion of mGluR1 leads to severe symptoms of ataxia [31]. The knockout of TRPC3 causes a less severe phenotype than that of mGluR1 but motor coordination deficit is clearly evident [3]. In humans, motor coordination is strongly impaired by autoantibodies against mGluR1 [32]. Patients with a point mutation of *TRPC3* gene (R762H) show late-onset unidentified ataxia [33,34]. It has also been postulated that TRPC3 signaling is disrupted in spinocerebellar ataxia 1 (SCA1) disease [4]. Interestingly, autoantibodies against GABA_B_Rs have been found in an agrypnia patient showing ataxia [35]. Although no causal relationship has been established between the GABA_B_R antibodies and motor coordination impairment of this patient, the current finding that GABA_B_Rs play an important role in shaping the response of Purkinje neuron firing to mGluR1 activation, a process mediated by the TRPC3 channel, brings an intriguing possibility that disrupting GABA_B_R function may interfere with motor coordination through deregulation of mGluR1/TRPC3 mediated activities in this cell type. In addition to regulating Purkinje cell firing frequency, sEPSC is closely associated to cerebellar LTD, a long term synaptic plasticity change that underlies motor learning [12]. It is therefore speculated that the potentiation of mGluR1/TRPC3 activity by GABA_B_Rs can also impact motor learning. However, the more widespread expression of GABA_B_Rs and TRPC3 in other cell types in the cerebellum rather than just Purkinje neurons plus the presence of both pre- and postsynaptic effects of the GABA_B_R function make the investigation of GABA_B_R- and TRPC3-regulated processes in normal conditions and various diseases very challenging [4,18,19]. Nevertheless, the finding that GABA_B_Rs and mGluR1 converge onto the TRPC3 channel in postsynaptic transmission in Purkinje neurons should open a new avenue to help improve cerebellar functions, such as motor coordination and learning, and combat cerebellar diseases, for instance the various types of ataxia. 

## 5. Conclusions

We conclude that TRPC3 is responsible for the potentiation of mGluR1 activation-evoked sEPSC by GABA_B_Rs at the parallel fiber-Purkinje cell synapses in the cerebellum and this regulation helps put a timed break on the sEPSC-induced increase in Purkinje cell firing.
